# Cutaneous manifestations-associated with tuberous sclerosis complex and the use of topical rapamycin in the United States: a sub-analysis of an international survey of caregivers and patients

**DOI:** 10.1186/s13023-025-03653-z

**Published:** 2025-08-25

**Authors:** Sreedevi Boggarapu, Gabrielle Rushing, Ashley Pounders, Steven L. Roberds, Eric Beresford

**Affiliations:** 1 Nobelpharma America, LLC. 3, 4520 East-West Highway, Suite 400, Bethesda, MD 20814 USA; 2 TSC Alliance, Silver Spring, MD USA

**Keywords:** Facial angiofibroma, Mechanistic target of rapamycin, Surgical removal, Topical rapamycin, Tuberous sclerosis complex

## Abstract

**Background:**

This analysis was aimed to characterize cutaneous manifestations associated with tuberous sclerosis complex (TSC) and management of facial angiofibroma in the United States from a patient/caregiver perspective. Data was collected from an international survey of TSC Alliance conducted during May-June 2017 by distributing a link to patients/caregivers through various channels including social media.

**Results:**

Of the 418 caregivers and 133 patients, 336 (80.0%) caregivers and 98 (73.7%) patients reported cutaneous manifestations. Increased incidence in cutaneous manifestations was observed with age with the highest incidence in the age group spanning 27–45 years. More than half of the responders reported minor, moderate or major changes to their lifestyle because of the impact of cutaneous manifestations on the quality of life. The presence of other TSC-related manifestations studied in this survey (epilepsy, non-malignant brain tumours, developmental delay, learning or memory issues, kidney issues, communication issues, behavioural issues, sleep problems, anxiety or depression, heart issues, eye issues, dental issues, bone or skeletal issues, lung issues, and liver or pancreatic issues) was significantly higher in patients with cutaneous manifestations. Surgical removal was reported by 28.6% caregivers and 61.2% patients. Compounded topical rapamycin use for the management of facial angiofibroma was reported by 31.3% caregivers/23.5% patients, out of whom, improvement in skin condition was reported by 64.8% caregivers/69.6% patients. Overall, 82.9% of caregivers and 73.9% patients reported improvement as moderately effective or very effective.

**Conclusions:**

In patients with cutaneous manifestations, a higher frequency of other TSC manifestations was observed. Presence of cutaneous manifestations impacted the quality of life of more than half of the responders. Surgical removal of cutaneous manifestations and compounded topical rapamycin treatment for the management of facial angiofibroma were reported. Compounded topical rapamycin use for the management of facial angiofibroma was reported as moderately effective or very effective by most of the responders.

## Introduction

Tuberous sclerosis complex (TSC) is a rare autosomal dominant genetic disorder with an estimated prevalence of 1 in 5,800 live births [1, 2]. Loss of function mutations in *TSC1* or *TSC2* genes encoding hamartin and tuberin, respectively, cause TSC by interfering with the cell’s ability to negatively regulate the mechanistic target of rapamycin (mTOR) pathway [1, 3, 4]. TSC is characterized by the presence of hamartomas, non-malignant tumors, and other lesions in multiple organs, predominantly on the skin and in the brain, kidneys, lungs, and heart that can lead to organ dysfunction [1].

Cutaneous manifestations such as facial angiofibroma, ungual fibroma, hypopigmented macules, fibrous cephalic plaques and shagreen patches can affect > 90% of patients with TSC [5]. Physical removal of facial angiofibroma with laser and microdermabrasion is in clinical practice [4, 6]. However, clinical outcomes of these treatments have been unsatisfactory due to pain, hyperpigmentation, scarring, bleeding, and recurrence of lesions [4]. Use of systemic mTOR inhibitors have shown ancillary improvement of facial angiofibroma during therapy for other manifestations [7, 8].

Topical mTOR inhibitors reduced the size and improved the color of facial angiofibroma in multiple clinical trials and case reports [9–15], which was maintained over time [15–17], and was further confirmed in a post marketing surveillance conducted in Japan [18]. In theUnited States , ~ 25% of patients with facial angiofibroma were reported to use compounded topical rapamycin as the treatment and < 20% patients received laser or abrasive treatments based on data analysis from the TSC Alliance Natural History Database conducted before the approval of topical sirolimus 0.2% gel [19].

An international survey was conducted in 2017 by the TSC Alliance to understand the perspectives of patients with TSC or their caregivers as part of an externally led patient focused drug development meeting (PFDD). In this article, we present the sub-analysis of data on TSC cutaneous manifestations and treatment trends for facial angiofibroma in the United States, focusing on topical rapamycin.

## Methods

The TSC Alliance conducted an international drug development survey between 15th May 2017 to 14th June 2017 in English, Spanish, and French, which was made available to patients and caregivers through various channels including social media. A total of 1,309 survey responses were received from patients with TSC or their caregivers, or from patients with sporadic lymphangioleiomyomatosis (LAM) representing 57 countries, with the United States (66.5% of responders) being the most well-represented country. Data from 418 caregivers and 133 patients with TSC who responded from the United States was used for the analysis. The survey was designed to understand the perspectives of patients with TSC or their caregivers regarding the impact of TSC manifestations on daily lives, the benefits and limitations of existing treatments, and their priorities for new treatments. The survey included 26 questions for caregivers answering on behalf of dependent patients and 21 questions for patients with TSC aged 13 years or older who were able to answer for themselves [20]. This sub-analysis of the survey is focused on the characterization of cutaneous manifestations of TSC in the United States including the data of responders on the English version.

The subgroup analysis included the variables of age at time of survey, age at time of TSC diagnosis, and the presence and number of TSC-related manifestations reported for each individual. Data were collected on 17 TSC-related manifestations including epilepsy, developmental delay or intellectual disability, sleep issues, behavioral or social issues, cutaneous manifestations (including facial angiofibroma) communication problems, learning or memory issues, anxiety and depression, kidney issues, nonmalignant brain tumors, heart issues, dental issues, eye issues, liver/pancreas issues, bone or skeletal issues, lung issues, and lymphatic issues.

No protected health information was collected. Age of patients at the time of the survey and at the time of TSC diagnosis were collected as the following age groups: 0–23 months, 2–5 Years, 6–12 years, 13–17 years, 18–26 years, 27–45 years, and ≥ 46 years. Impact of cutaneous manifestations on lifestyle was collected as degree of change in lifestyle due to cutaneous manifestation using the following scale: no change, minor change, moderate change, and severe change. Caregiver responses for the impact of cutaneous manifestations were analyzed by the age groups of patients (≥ 13 years or < 13 years), enabling comparison to the group of patients ≥ 13 years old who were able to answer for themselves. Data was collected on the use of topical rapamycin for the management of facial angiofibroma and procedures for surgical removal of cutaneous manifestations (laser therapy, microdermabrasion, etc.). The use of topical rapamycin was analyzed by age groups reported at the time of the survey. Data collected on oral mTOR inhibitor use for the management of TSC manifestations (but not specific to cutaneous manifestations) were included in the analysis because they could improve the skin condition [7, 8]. The patient- or caregiver-reported effectiveness of topical rapamycin and improvement of cutaneous condition with topical and oral mTOR inhibitors were analyzed.

### Statistical analysis

Counts and frequency were reported for all categorical variables. Statistical analyses for comparisons between groups were performed using chi-square tests or Fisher Exact tests if n values ≤ 5. The p-value ≤ 0.05 was considered statistically significant. Statistical analysis was performed using R studio version 1.1.383.

## Results

### Demographics

Of the 418 caregivers and 133 patients with TSC who responded from the United States using the English version of survey, 336 (80.4%) caregivers and 98 (73.7%) patients with TSC reported cutaneous manifestations. 11 (0.03%) caregivers were taking care of 2 individuals and 15 (0.04%) cared for ≥ 3 individuals.

The prevalence of cutaneous manifestations increased with age with the highest in the 27–45 years age group as reported by caregivers and patients (Table 1). Most patients (> 74%) were diagnosed with TSC within 2 years of birth as reported by caregivers while patients reporting for themselves tended to be diagnosed later in life (Table 2).

**Table 1:** Patients age at the time of survey as reported by caregivers and patients by the presence of cutaneous manifestations

Parameter	Reported by Caregivers		Reported by Patients	
	Cutaneous manifestations		Cutaneous manifestations	
	Presence *N* = 336 n (%)	Absence *N* = 82 n (%)	Presence *N* = 98 n (%)	Absence *N* = 35 n (%)
0–23 months	22 (6.5)	18 (22.0)		
2–5 years	45 (13.4)	19 (23.2)		
6–12 years	60 (17.9)	14 (17.1)		
13–17 years	49 (14.6)	12 (14.6)	1 (1.0)	2 (5.7)
18–26 years	70 (20.8)	3 (3.7)	8 (8.2)	5 (14.3)
27–45 years	76 (22.6)	11 (13.4)	54 (55.1)	14 (40.0)
≥ 46 years	16 (4.8)	4 (4.9)	35 (35.7)	14 (40.0)

Data are n (%)

**Table 2:** Patients age at the time of TSC diagnosis as reported by caregivers and patients

Parameter	Reported by Caregivers		Reported by Patients	
	Cutaneous manifestations		Cutaneous manifestations	
	Presence *N* = 336	Absence *N* = 82	Presence *N* = 98	Absence *N* = 35
0–23 months	252 (75.0)	61 (74.4)	20 (20.4)	2 (5.7)
2–5 years	40 (11.9)	13 (15.9)	16 (16.3)	4 (11.4)
6–12 years	23 (6.8)	3 (3.7)	11 (11.2)	4 (11.4)
13–17 years	13 (3.9)	2 (2.4)	10 (10.2)	4 (11.4)
18–26 years	5 (1.5)	1 (1.2)	15 (15.3)	6 (17.1)
27–45 years	2 (0.6)	1 (1.2)	20 (20.4)	11 (31.4)
≥ 46 years	1 (0.3)	1 (1.2)	6 (6.1)	2 (5.7)

Data are n (%)

## TSC-related manifestations

**Fig. 1:**
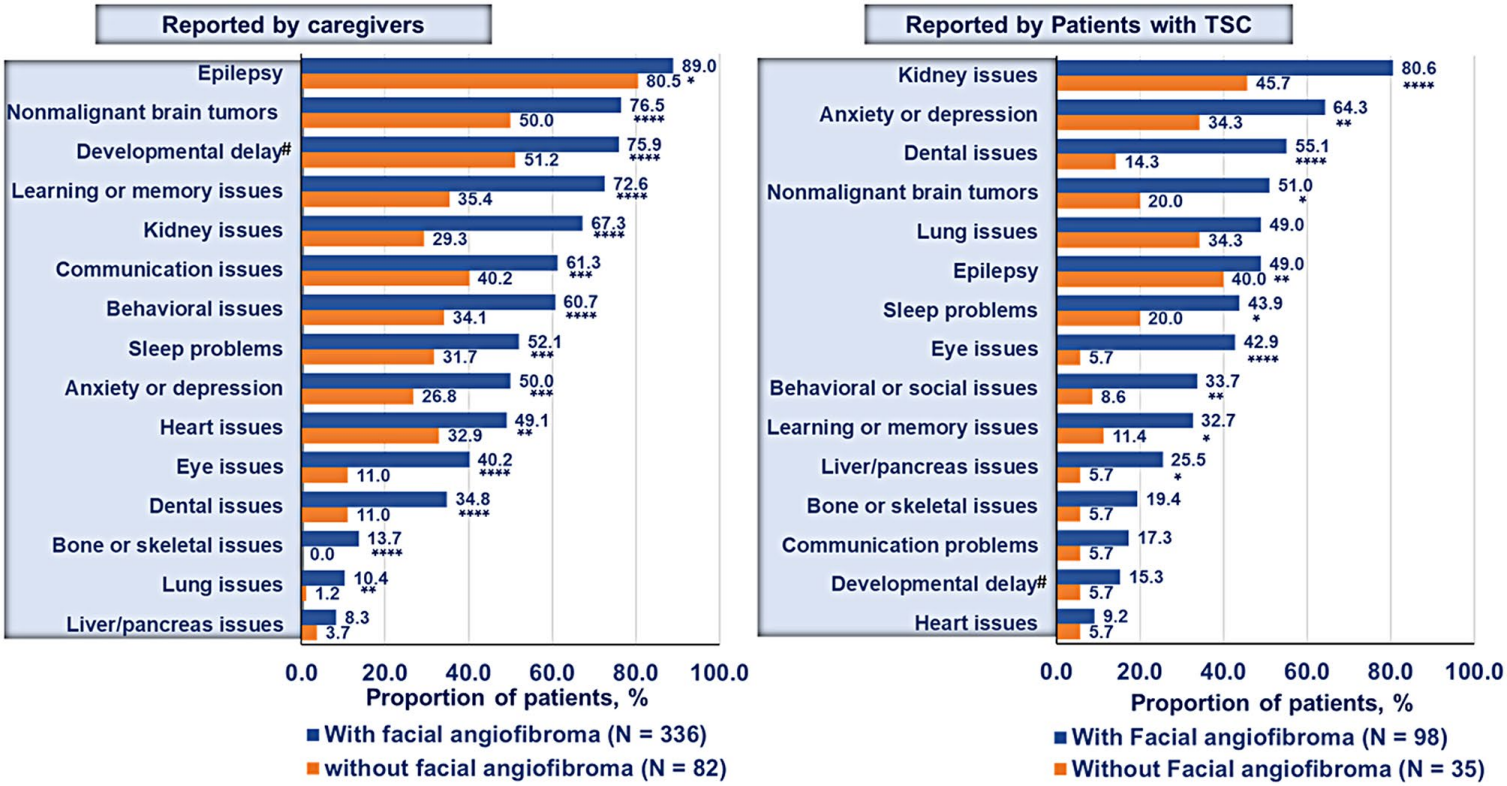
TSC-related manifestations in patients with cutaneous manifestations versus without cutaneous manifestations as reported by (**A**) caregivers and (**B**) patients. ^#^developmental delay/intellectual disability; *p* < 0.05; **<0.01; ***p ≤ 0.001; ****p < 0.0001 comparing patients with versus without cutaneous manifestations. TSC, tuberous sclerosis complex

The presence of most TSC-related manifestations was significantly higher in patients with cutaneous manifestations as reported by caregivers and patients (Fig. 1).

On average, the number of TSC-related manifestations coexisting in a patient was significantly higher (*p* < 0.0001) in patients with cutaneous manifestations than patients without cutaneous manifestations as reported by caregivers (9.6 vs. 5.4) and self-reported by patients (7.9 vs. 3.6). The number of TSC-related manifestations varied widely ranging from 1 to 18 manifestations observed per individual.

## Impact on lifestyle

**Fig. 2:**
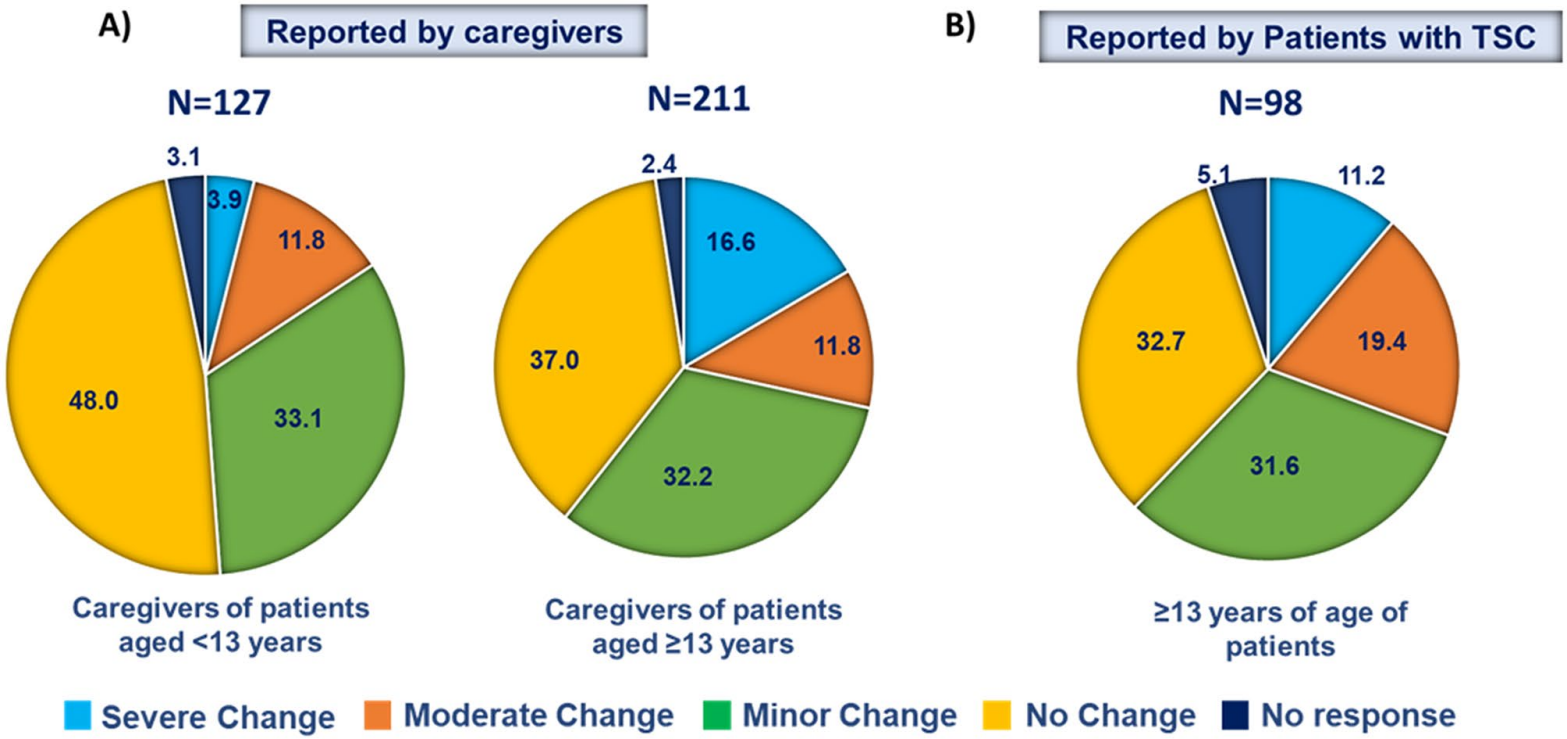
Impact of cutaneous manifestations associated with TSC on the lifestyle of (**A**) caregivers and (**B**) patients. TSC, tuberous sclerosis complex

More than half of both caregivers and patients reported a minor, moderate, or major changes to their lifestyle impact of cutaneous manifestations on lifestyle. A sub-analysis showed a higher proportion of caregivers who responded on behalf of patients aged ≥ 13 years (16.6%) vs. < 13 years (3.9%) reported a major change to their lifestyle due to TSC cutaneous manifestations (Fig. 2) while 11.2% of patients self-reported a severe impact on lifestyle (Fig. 2).

### Treatment approaches

**Fig. 3:**
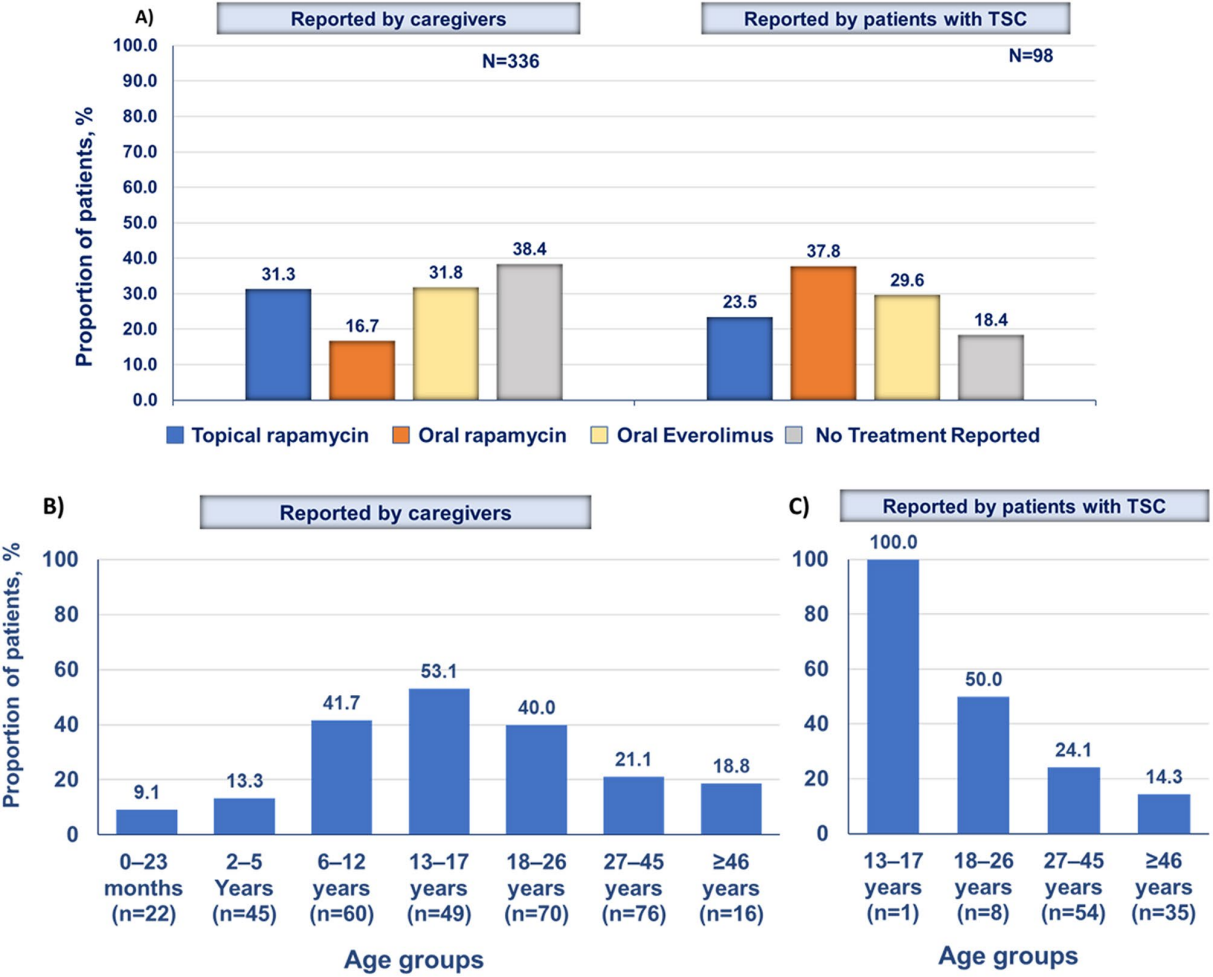
Treatments for the management of facial angiofibroma as reported by caregivers and patients (**A**) Subgroup analysis of topical rapamycin use by agegroups as reported caregivers (**B**) and patients (**C**). TSC, tuberous sclerosis complex

Surgical removal of cutaneous manifestations was reported by 96 (28.6%) caregivers and 60 (61.2%) patients. Despite the lack of FDA-approved topical mTOR inhibitor in the United States at the time of survey, use of compounded topical rapamycin for the management of facial angiofibroma was reported by 105 (31.3%) caregivers and 23 (23.5%) patients with TSC (Fig. 3A). Use of systemic mTOR inhibitors that include oral rapamycin/sirolimus or everolimus was reported by 16.7% and 31.8% caregivers, respectively, and 37.8% and 29.6% patients with TSC, respectively (Fig. 3A). Use of ≥ 1 mTOR inhibitors that includes systemic or topical rapamycin or everolimus was reported by 191 (56.8%) caregivers and 64 (65.3%) patients. A total of 129 (38.4%) caregivers and 18 (18.4%) patients did not report use of topical/oral mTOR inhibitors or physical treatment.

A subgroup analyses by age groups showed that 13–17 years age group, followed by 6–12 years age group had a higher rate of topical rapamycin use as reported by caregivers (Fig. 3B). Self-reports by patients revealed a decreasing trend of topical rapamycin use with age; however, the n values were very low (Fig. 3C).

**Fig. 4:**
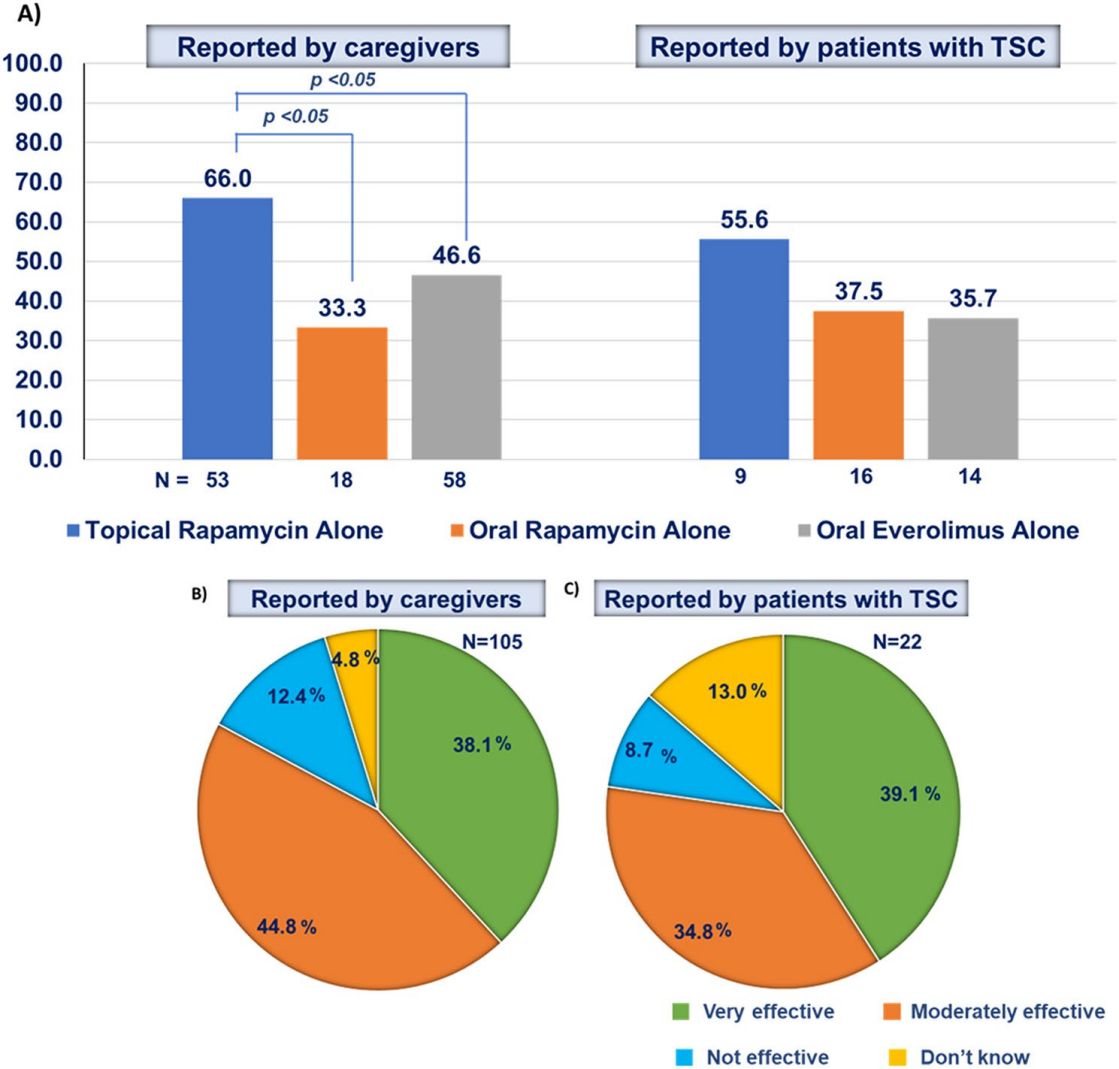
Improvement in facial angiofibroma after using topical or oral mTOR inhibitors as reported by caregivers and patients (**A**) and effectiveness of topical rapamycin as reported by caregivers (**B**) and patients (**C**). TSC, tuberous sclerosis complex

Overall, a higher proportion of patients using topical rapamycin reported improvement in facial angiofibroma compared with the use of oral rapamycin or everolimus as reported by caregivers (64.8%, 55.4%, and 57.0%, respectively) and patients with TSC (69.6%, 54.1%, and 48.3%, respectively). A statistically significant difference was observed for the comparison of the improvement in facial angiofibroma of topical rapamycin alone versus oral rapamycin or everolimus alone as reported by caregivers (Fig. 4A). Topical rapamycin use for the management of facial angiofibroma was reported to be moderately effective or very effective by 82.9% of caregivers and 73.9% of patients with TSC (Fig. 4B, C).

## Discussion

The sub-analysis of the international survey was conducted to characterize TSC cutaneous manifestations and understand the current treatment approaches for the management of facial angiofibroma in the United States. The presence of other TSC manifestations was significantly higher in patients with cutaneous manifestations compared with patients without cutaneous manifestations. In addition, the number of TSC manifestations coexisting in a patient was significantly higher in patients with cutaneous manifestations. Use of topical rapamycin treatment for the management of facial angiofibroma was reported by 31.3% of caregivers and 23.5% of patients with TSC. Improvement in cutaneous manifestations was more frequently reported with topical rapamycin treatment compared with other treatment approaches. The majority of caregivers and patients reported topical rapamycin as moderately effective or very effective.

Facial disfigurement due to angiofibroma can negatively impact the health-related quality of life (HRQoL) of patients and their caregivers, which can lead to negative psychosocial outcomes. Though the neurological and psychiatric symptoms are the most burdensome manifestations of TSC for caregivers, the panelists and participants of Patient-Focused Drug Development (PFDD) meeting sponsored by the TSC Alliance mentioned that facial angiofibromas are often the most visible sign of TSC and may be the first symptom leading to a diagnosis for mildly affected patients [20]. Facial angiofibroma poses physical as well as psychological risks. Due to bleeding of facial angiofibroma, one of the participants recalled being removed from the sports to avoid infection. Participants also mentioned that patients were teased for the bumps on their faces [20].

In a study by Crall et al., the quality of life of patients who were receiving treatment for the management of facial angiofibroma had significantly better HRQoL scores as measured by the Children’s Dermatology Life Quality Index (CDLQI; mean 3.8 vs. 9.5, *p* = 0.001) compared with those who were not receiving treatment [21]. More than half of the caregivers and patients with TSC in our study reported that cutaneous manifestations impact their lifestyle which requires minor, moderate, or severe changes.

Facial angiofibroma requires chronic treatment throughout life, however, current options in the United States are limited, and the clinical outcomes are generally unsatisfactory. Physical removal of cutaneous manifestations is difficult as the lesions involve the entire dermis. Several caregivers and adults with TSC who participated in the PFDD meeting recalled the pain and discomfort of facial angiofibroma removal procedures [20]. One of the participants who was treated by a dermatologist as a child in the late 1970s recalled periodic visits to “*try the newest torture therapy to remove the angiofibromas on my nose and cheeks*” [20].

Systemic mTOR inhibitors are used to treat multiple manifestations of TSC [22–26], however, their use for the treatment of facial angiofibroma specifically would be off label. Systemic exposure to mTOR inhibitors can be associated with serious side effects, most of which are associated with their immunosuppressive action [27]. Treatment with oral mTOR inhibitors was reported by caregivers and patients with TSC, which is likely attributed to the presence of other conditions such as subependymal giant cell astrocytoma, renal angiomyolipoma, pulmonary lymphangioleiomyomatosis, and focal-onset seizures associated with TSC for which use of oral mTOR inhibitors is approved; this use potentially contributes to the improvement of facial angiofibroma [28, 29].

Topical rapamycin was being prescribed as a compounded formulation of cream/ointment at the time of survey [21]. Most responders in this study rated the effectiveness of topical rapamycin as moderately or highly effective. However, due to their compounded production, these treatments can vary widely in dosage (0.03–1.00%), excipients, and cost, posing a huge unmet need for a standardized and accessible product (20, 30–31). A few comments on the international drug development survey indicated when specialty pharmacies were not an option, parents would mix oral sirolimus into a topical formulation themselves [20].

Topical sirolimus, a non-invasive treatment option for the treatment of facial angiofibroma is beneficial [9–18] and further expands treatment access to the broader population of those affected by TSC including individuals with autism and/or developmental delay that cannot utilize laser treatment. In addition, avoiding the use of anesthesia is beneficial as TSC patients are commonly sedated for imaging. Wheless et al. reported low systemic absorption of sirolimus further supporting topical formulation as the safest option compared with oral mTOR inhibitors for treating facial angiofibromas [16]. This is particularly helpful for patients who are on a transplant list or have had a lung or kidney transplantation, as the transplant medications are not required to be adjusted.

Early treatment with sirolimus gel may maintain the skin at near-normal levels in children with TSC, which further emphasizes the unmet need to access the treatment [32].

This major limitation of this analysis is that the international drug survey was designed to understand patient perspectives regarding all TSC-related manifestations, but it was not specific to cutaneous manifestations. This was not designed to collect data prospectively and is biased based on who was notified of the availability of the survey and chose to respond.

## Conclusions

The majority of patients represented in this survey were diagnosed with TSC within 0–23 months of age. Patients with cutaneous manifestations were relatively older than the patients without cutaneous manifestations. The presence of other TSC manifestations in patients with cutaneous manifestations were significantly higher compared with patients without cutaneous manifestations. In patients with cutaneous manifestations, a higher frequency of other TSC manifestations was observed, illustrating the importance of accurate diagnosis of cutaneous manifestations by dermatologists and referral to a comprehensive multi-disciplinary TSC Clinic for surveillance and management of TSC manifestations. Use of topical rapamycin for the management of facial angiofibroma was reported by about one-third of the survey responders, despite the lack of an approved topical rapamycin formulation at the time of survey administration. Improvement in facial angiofibroma was more common with topical rapamycin than the other treatment approaches, and most (> 70%) caregivers and patients rated topical rapamycin as moderately effective or very effective. Recently, the FDA approved topical rapamycin 0.2% gel for the treatment of TSC facial angiofibroma.

## Data Availability

All data requests should be submitted to the corresponding author for consideration. Access to available anonymized data may be granted following review.
